# Supplementary material for: Convergence in phosphorus constraints to photosynthesis in forests around the world
Source: Nat Commun. 2022 Aug 25;13:5005. doi: 10.1038/s41467-022-32545-0 (PMC9411118; doi:10.1038/s41467-022-32545-0)
Supplement: Supplementary file 1 — Supplementary Information [file 41467_2022_32545_MOESM1_ESM.pdf]

# Supplementary materials for

## "Convergence in phosphorus constraints to photosynthesis in forests around the world"

### Supplementary Note 1: Statistical analyses of N and P dependencies of photosynthesis and mass- and area-basis

In the main analysis, we use simple ordinary least-squares (OLS) and multiple regressions to find a model that involves both N and P dependencies of photosynthesis and the biochemical terms $V_{cmax}$ and $J_{max}$ on a mass-basis. In the main text and results (Figure 1, Table 1) we distinguished species between "low P" or "moderate P" classes based on a threshold for their leaf P concentration (greater or less than 0.92 mg $g^{-1}$), showing that the response of photosynthetic traits to leaf N was steeper for moderate-P species than those with low P concentrations (Supplementary Table 3). In those analyses we most strongly emphasised analyses concerning photosynthetic characteristics and N and P concentrations, all expressed on a per dry mass basis. Here we discuss alternative analyses that support the main conclusions, including area-based expressions. Each major section below highlights an additional supporting analysis. The additional analyses we present further demonstrate the robust nature of the main results.

**Mass-basis traits**

For models with the mass-based photosynthetic characteristics ($V_{cmax\_mass}$ or $J_{max\_mass}$ or $A_{mass}$) treated as a function of leaf N, P, and the N × P interaction, the interaction term was positive and clearly significant in all cases ($P < 0.05$, Table 2). This indicates that the response of $V_{cmax\_mass}$ or $J_{max\_mass}$ or $A_{mass}$ to leaf N is steeper at higher leaf P and, equally, that the responses to leaf P are steeper at higher leaf N. Whole-model goodness-of-fit ($r^2$) values for $V_{cmax\_mass}$ were 0.43; for $J_{max\_mass}$, 0.46; for $A_{mass}$, 0.35 (Table 2).

**Area-basis traits**

For models with the photosynthetic trait ($V_{cmax}$ or $J_{max}$ or $A_{net,a}$) treated as a function of N, P, and N × P interaction, this interaction was clearly non-significant in all cases (for $V_{cmax}$ and $J_{max}$, P > 0.4; for $A_{net,a}$, $P = 0.39$; Table 2). We analysed these models with the photosynthetic parameters as a function of N and P only, and the coefficient for P was significant in all cases (all $P < 0.005$) indicating that, at any given leaf N, $V_{cmax}$, $J_{max}$ and $A_{net,a}$ were all higher at higher leaf P. Furthermore, in each case the explanatory power (indicated by regression $F$ value) associated with leaf P was substantially higher than that for leaf N. For $A_{net,a}$ and $V_{cmax}$ the F-value for the leaf P term was 4-5-fold higher than that for leaf N, and for $J_{max}$ it was 18-fold higher.

The whole-model $r^2$ values were notably lower for area-basis relationships than for mass-basis relationships. For $V_{cmax}$, $r^2 = 0.18$, for $J_{max}$, $r^2 = 0.22$, and for $A_{net,a}$, $r^2 = 0.07$ (Supplementary Table 4). This pattern of describing less variation for the area-based versus mass-based relationships is standard and well understood for analyses of leaf physiological traits[S1,S2]. The drawback of area-basis relationships is that they are confounded with variation in $M_a$[S2,S3]. For example, a gradient of increasing $A_{net,a}$ among species by rank will generally

be driven both by increasing photosynthetic capacity as well as by increasing $M_a$, especially from an increased depth of mesophyll layers as a consequence of thicker leaves tending to have greater $M_a$. The same is true of gradients in leaf $N_{area}$ or leaf $P_{area}$. Hence, observed relationships between $A_{net,a}$ and leaf nutrients on an area basis tend to be rather weak (Supplementary Table 3). Conversely, mass-basis relationships incorporate the strong covariance between $M_a$ and leaf nutrient concentrations, meaning that a tight relationship between, say, $A_{mass}$ and $N_{mass}$ reflects both the causal link between these traits, and the $M_a$ effect[S2]. A straightforward way to handle this linkage is to include $M_a$ as an additional term in the regression model so as to quantify the $M_a$ -independent contribution of leaf N (or P) to photosynthesis or, indeed, the $M_a$-independent contribution of the N × P interaction (Table 2). Further, the regression coefficient for $M_a$ in such a model indicates the N- or P-independent effect of $M_a$ on photosynthesis.

**Mass-basis traits with leaf mass per unit area considered**

For models with mass-basis photosynthetic parameters ($A_{mass}$, $V_{cmax\_mass}$ or $J_{max\_mass}$) treated as a function of N, P, N x P, and $M_a$, in each case $M_a$ showed a clear negative effect on photosynthetic traits, sometimes adding several percentage points to model explanatory power (whole-model $r^2$ for $J_{max\_mass}$, 0.55 compared to $r^2 = 0.48$ without $M_a$; for $A_{mass}$, $r^2= 0.46$ compared to $r^2 = 0.45$ without $M_a$; Table 2).

For the $V_{cmax\_mass}$ and $J_{max\_mass}$ models including $M_a$, the N × P interaction was still positive and highly significant (both P = 0.001). For the $A_{mass}$ model, the N × P interaction was still positive but considerably weaker (P = 0.033). Fitting that model without the N × P interaction term, the main effect of P was strong (P = 0.003) and positive, just as when $M_a$ was not included. However, the inclusion of $M_a$ rendered the leaf N effect non-significant ($P = 0.24$).

In summary, in all analyses for relationships involving the photosynthesis variables and leaf N and P, we observed a clear stimulatory effect of higher leaf P on photosynthetic processes or, equally, a clear limiting effect of low leaf P. This was observed variously as slope-shifts in $V_{cmax\_mass}$-N, $J_{max\_mass}$-N relationships (mass-basis results), or for the $V_{cmax}$-N, $J_{max}$-N or $A_{net,a}$-N relationships to have higher intercepts at higher leaf P (area-basis results). These patterns were equally clear when controlling statistically for $M_a$ covariation.

**Triose-P limitations in the FvCB photosynthesis model framework**

The specific mechanism by which low leaf P concentrations decrease photosynthesis are not well understood. However, beginning with Sharkey[S4,S5] it has been suggested that low cytosolic inorganic P ($P_i$) may decrease photosynthesis at very high [$CO_2$] by reducing the capacity for RuBP regeneration. To capture this effect, an additional term to the classic FvCB photosynthesis model was introduced[S4], involving triose-P (TP) limitations to photosynthetic metabolism. RuBP regeneration capacity may be limited by either the reductant produced through photosynthetic electron transport or by the regeneration of $P_i$. The new term for TP-limited photosynthesis was best associated with a lack of responsiveness to a drop in measurement $O_2$ partial pressure[25,S4]. Leaves are generally buffered from temporary drops in the $P_i$ available for metabolism by vacuolar $P_i$[S6]. Still, some researchers have construed TP limitations to arise from low P concentrations of leaves[S7]. For conventional field $A_{net} – C_i$ curve data it is difficult to fit TP limitation without additional measurements at low $O_2$ partial pressure[26]. In the pan-tropical species in our study, out of a total of 1631 $A_{net} – C_i$ curves, we could only successfully fit TP using conventional assumptions[26] for 48% of the curves. In those cases where we successfully fit a TP term, the effects of adding a new term for TP

limitation on the conventional fit for $J_{max}$ were small, about 2.4% overall. For the dataset where TP was determined with the P status classes in Fig. 1, the TP term was similar for low P concentration (TP = 8.5 ± 0.2 µmol $m^{-2}$ $s^{-1}$) as for TP for the moderate P status plants (TP = 9.2 ± 0.2 µmol m-2 s-1, mean ± standard error). The higher TP term for moderate P plants is consistent with the finding of higher TP limitation for plants with high P concentrations[25] in leaves compared to low P-plants.

With an inability to fit TP for the majority of the pan-tropical field dataset, a small impact of the new term on the fitted $J_{max}$, and very little difference in TP between different P status levels in the dataset, we conclude that TP limitation does not reflect low leaf P concentrations in the data analysed for the pan-tropical species here. Thus TP limitation did not influence our results, and overall has been misconstrued to be associated with low P concentrations.

**Supplementary references used in Supplementary Note 1**
(main references indicated by whole numbers)


S1. Reich, P. B. et al. Leaf structure (specific leaf area) modulates photosynthesis-nitrogen relations: evidence from within and across species and functional groups. Functional Ecology 12, 948-958, (1998).

S2. Rogers, A. et al. Improving representation of photosynthesis in Earth System Models. New Phytologist 204, 12-14, (2014).

S3. Osnas, J. L. D. et al. Global leaf trait relationships: mass, area, and the leaf economics spectrum. Science 340, 741-744, (2013).

S4. Sharkey, T. D. Photosynthesis in intact leaves of C3 plants - physics, physiology and rate limitations. Botanical Review 51, 53-105, (1985).

S5. Sharkey, T. D. Is triose phosphate utilization important for understanding photosynthesis? Journal of Experimental Botany 70, 5521-5525, (2019).

S6. Veneklaas, E. J. et al. Opportunities for improving phosphorus-use efficiency in crop plants. New Phytologist 195, 306-320, (2012).

S7. Campbell, C. D. & Sage, R. F. Interactions between the effects of atmospheric $CO_2$ content and P nutrition on photosynthesis in white lupin (*Lupinus albus* L.). Plant Cell and Environment 29, 844-853, (2006).

**Supplementary Figure 1. Hypothesised relationships between major biochemical variables for photosynthesis and leaf N and P.**

An idealized leaf photosynthetic ($A_{net}$) response to intercellular $CO_2$ ($C_i$) (solid black curve), showing the role of $V_{cmax}$ and $J_{max}$ in regulating $A$ (dashed curves for $A_v$ and $A_j$, respectively) in regulating the overall $A_{net}$ rate (Refs [26,30]). $A_{net}$ (solid black line) is the minimum of the rates $A_v$ and $A_j$, with the rate of respiration in the light ($R_l$) subtracted. We also provide an indication for how N and P might in turn regulate $V_{cmax}$ and $J_{max}$, respectively, based on the literature. Rubisco is Ribulose-bis-phosphate carboxylase, ATP is adenosine triphosphate, RuBP is Ribulose-2-phosphate, and $P_i$ is inorganic phosphate.

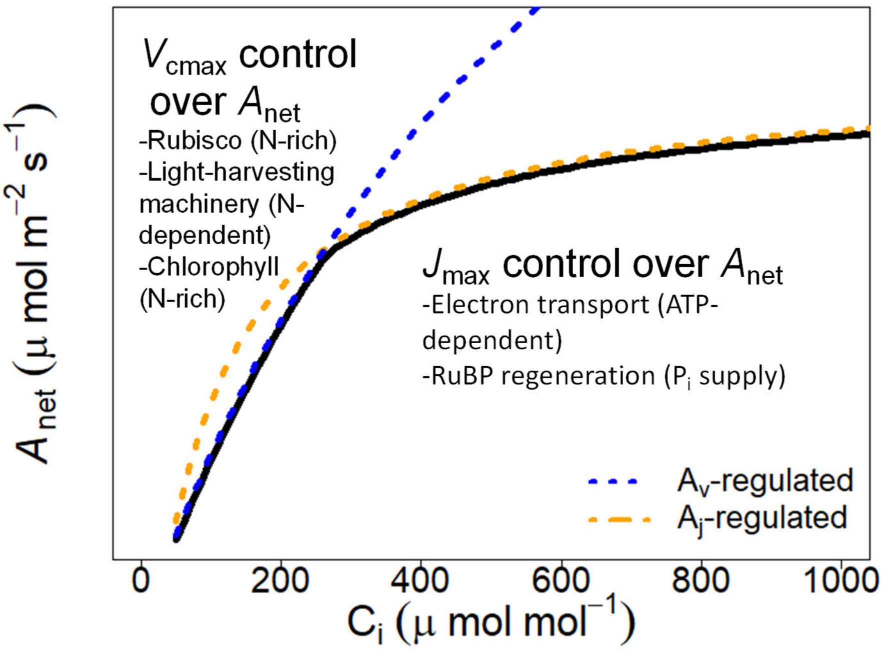

**Supplementary Figure 2. Results of relationships between biochemical variables for photosynthesis and leaf $P_{mass}$ from the literature.**

(a,b) $V_{cmax\_mass}$-$P_{mass}$ relationships and (c,d) $J_{max\_mass}$-$P_{mass}$ relationships for two different studies from the literature[29,50], each in comparison with the relationship from Fig. 1c,d ('This study'). $V_{cmax}$ and $J_{max}$ were normalized to 25 °C (see methods). Data shown as points are the species averages from each of the studies in the literature, with black lines and grey zones indicating the least-squares regression fits based on these points and 95% CIs, respectively. The cyan line indicates the relationship from this study (less the point data shown in each panel) and the cyan shaded zone is its 95 % CI. The extent of the lines delineates the range of the data, which is 50-fold for this study compared to 10-fold or less for the literature studies.

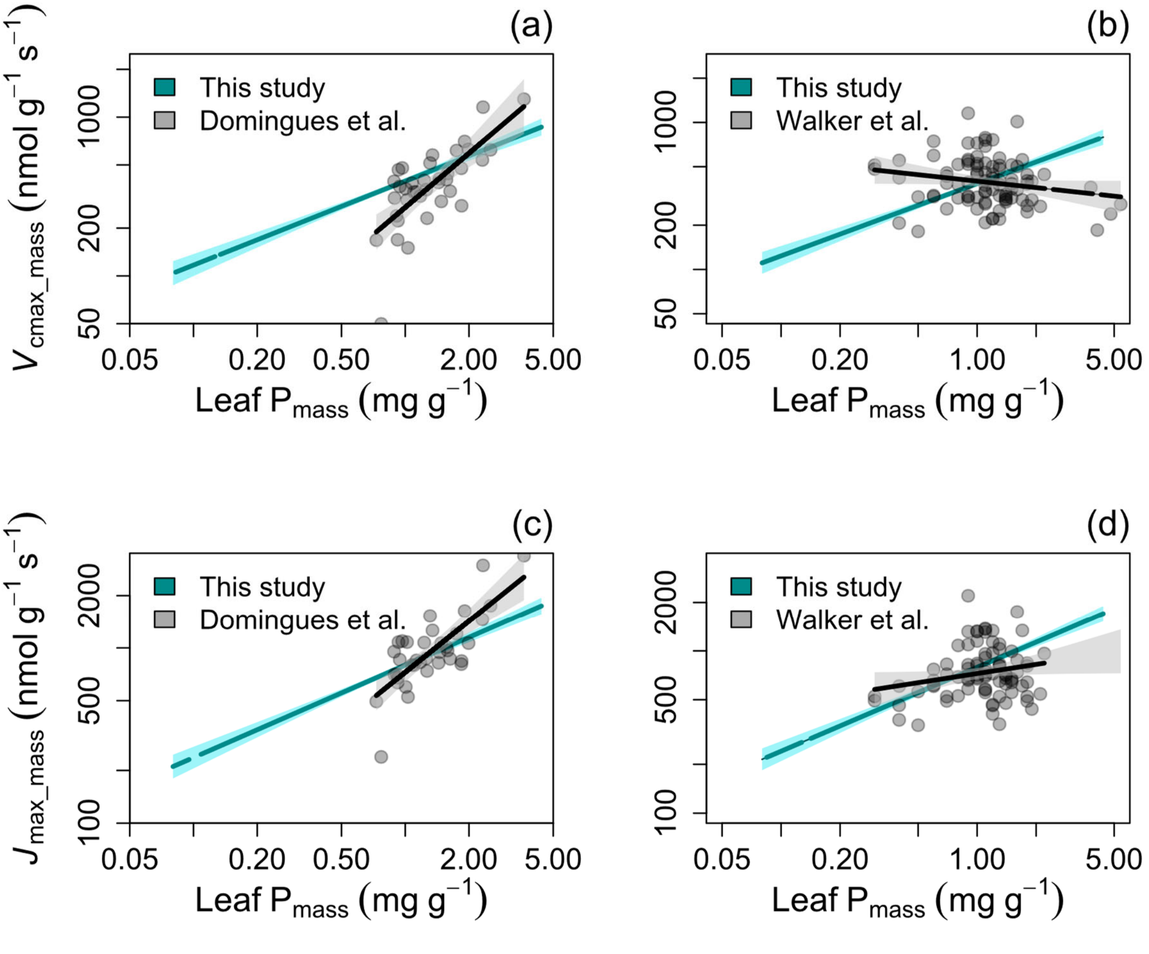

**Supplementary Figure 3. Geographical distribution of study sites in the analysis.**

Each study site is shown as a red point overlying a false-colour satellite image (Map data ©2022 Google). Some study sites are obscured because they are falling close to another site. In total there are 52 separate sites (Supplementary Table 1).

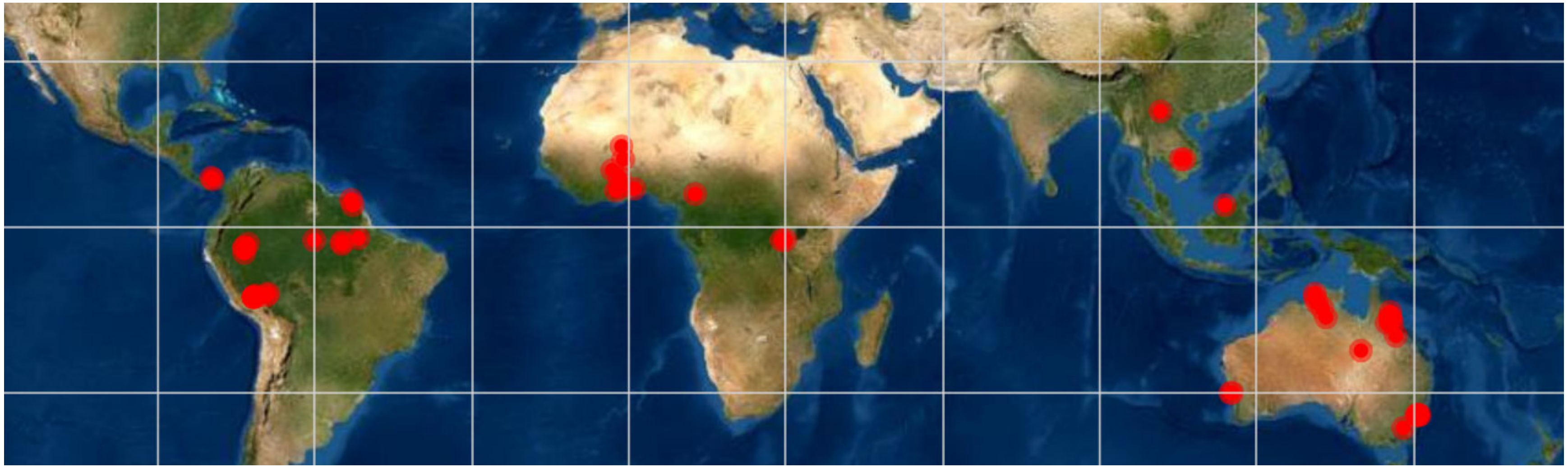

**Supplementary Figure 4. A circular 'tree of life' phylogenetic map of the angiosperm families represented in this study.**

Stems of the circle that are red show the families represented in the dataset, with families listed by continent in Supplementary Table 2.

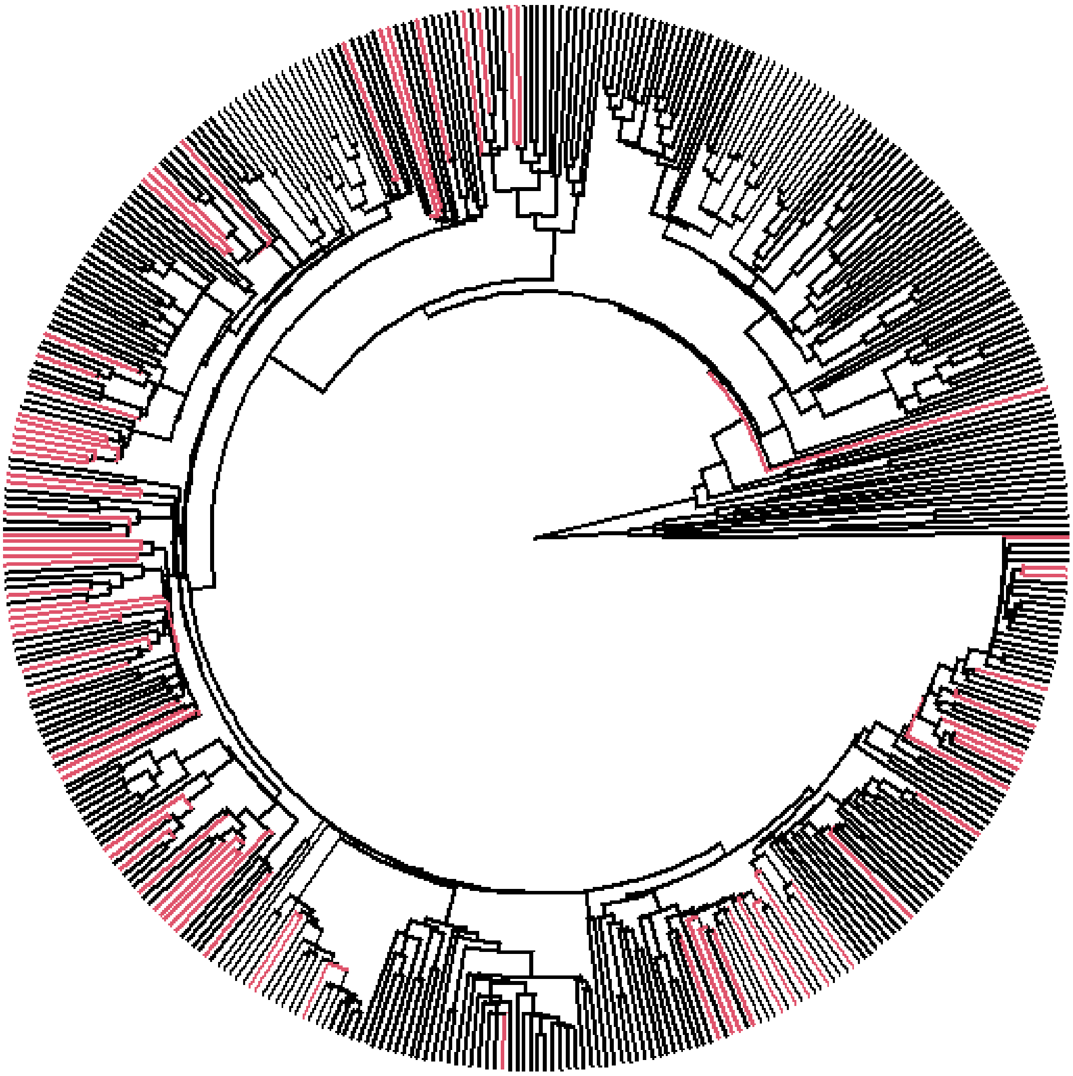

**Supplementary Figure 5. Effect of variation in the $P_{mass}$ threshold between moderate and low P on slopes.**

The photosynthesis dataset was divided between moderate and low P based on the threshold value, and the resultant slopes of $V_{cmax\_mass}$ as a function of $N_{mass}$, and $J_{max\_mass}$ as a function of $N_{mass}$, are shown. The default threshold value used for the main analysis in Table 1 was a $P_{mass}$ of 0.92 mg $g^{-1}$.

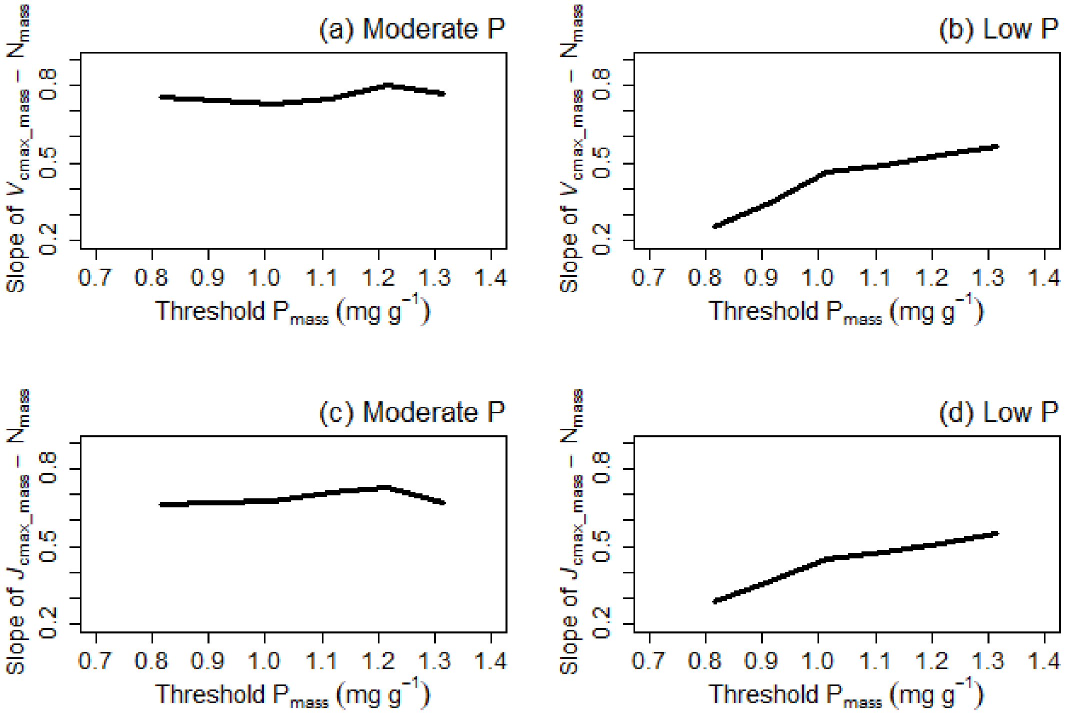

**Supplementary Figure 6. Scatter plots of $V_{cmax\_mass}$ and $J_{max\_mass}$ according to site climate.**

$V_{cmax\_mass}$ (a-c) and $J_{max\_mass}$ (d-f) normalized to 25 °C are shown as a function of site mean climate variables. Shown are mean annual temperature (a, d), mean annual precipitation (b, e) and aridity index determined according to the FAO (c, f). Symbols are coded by different shapes for different continents, and with colours corresponding to leaf P classes as described in Fig. 1 (e.g., "low P" points are purple and "moderate P" are dark grey). None of the relationships shown were statistically significant (P > 0.10).

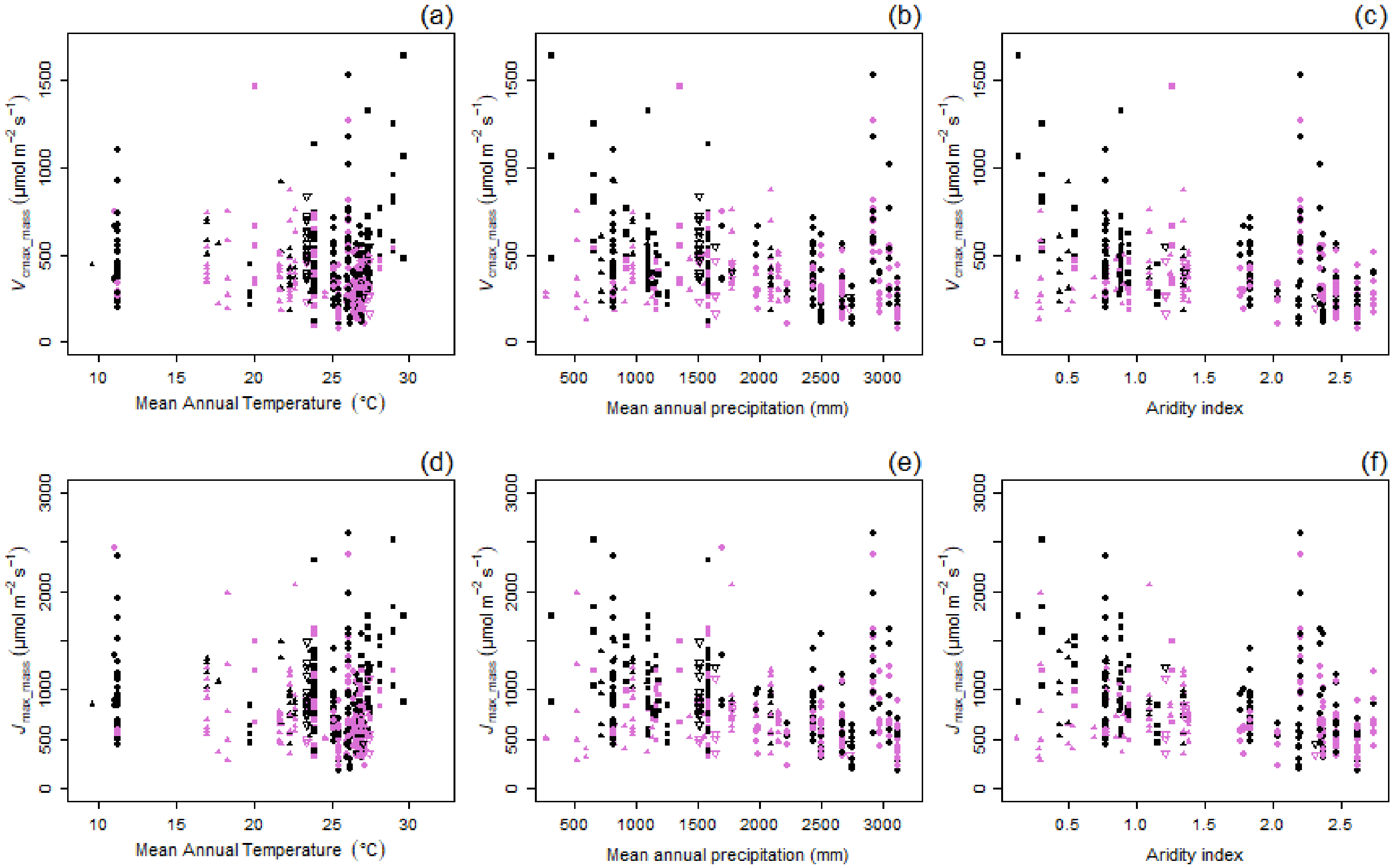

**Supplementary Figure 7. Northern hemisphere data for $V_{cmax\_mass}$-$P_{mass}$ and $J_{max\_mass}$-$P_{mass}$ compared with those in Fig. 1c,d.**

Relationships between (a) $V_{cmax\_mass}$-$P_{mass}$ and (b) $J_{max\_mass}$-$P_{mass}$ for four *Quercus* and *Pinus* species from the northern hemisphere (red points, normalized to 25 °C), and 38 species from the TRY v.5 database[44] (orange points, not temperature-normalised) compared with the overall relationships from the four continents from Fig. 1c, d. The overall relationships for this study are shown in blue with a shaded 95% CI area. Species from North America and Europe are: *P. taeda* (USA), *P. sylvestris* (Estonia) and *Q. virginiana* (USA) and *Q. robur* (UK). The points for northern hemisphere species have a narrow range of leaf $P_{mass}$ relative to the species from the other four continents. These points fall within the scatter for the other continents, but the overall relationships are not significant.

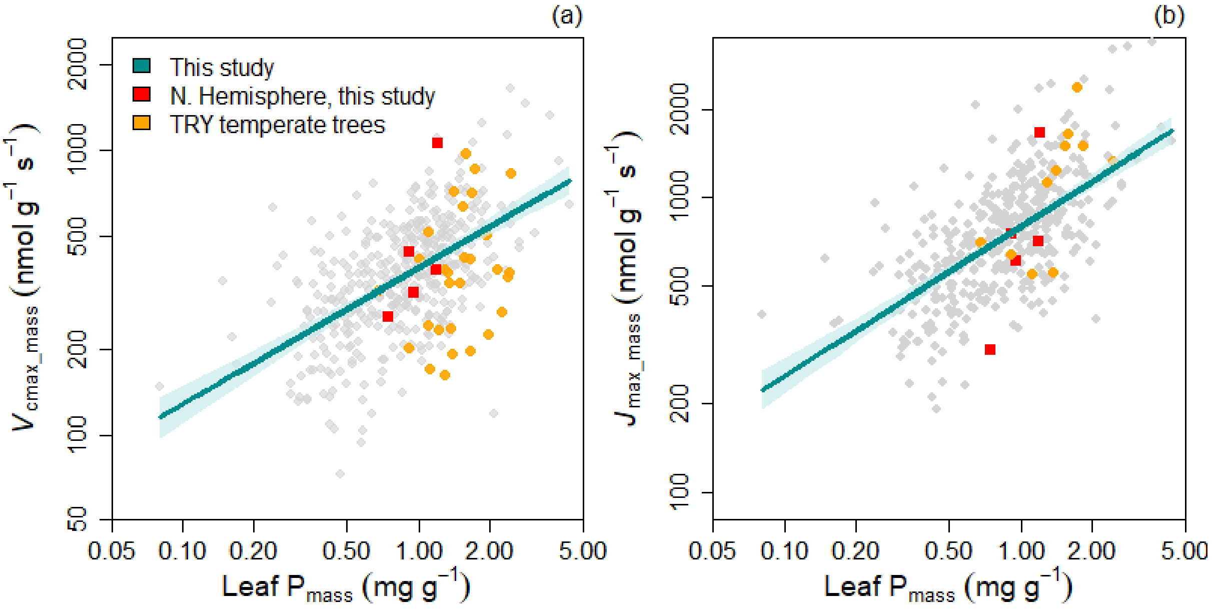

**Supplementary Figure 8. Three-dimensional surfaces for leaf photosynthetic biochemistry in relation to leaf N and leaf P concentrations.**

(a) Leaf carboxylation capacity ($V_{cmax\_mass}$) vs. leaf N concentration ($N_{mass}$) and leaf P concentration ($P_{mass}$) using multiple regression involving $N_{mass}$ and $P_{mass}$ with $N_{mass}$ * $P_{mass}$ interaction. The colour grid surface represents the least-squares solution: $V_{cmax\_mass}$ = exp(4.636 + 0.453*ln($N_{mass}$) - 0.546*ln($P_{mass}$) + 0.321*(ln($N_{mass}$) * ln($P_{mass}$))), $r^2$ = 0.42 (Table 2). Colour bands comprise a range of $V_{cmax\_mass}$ values. (b) Leaf RuBP regeneration capacity ($J_{max\_mass}$) vs. $N_{mass}$ and $P_{mass}$ using multiple regression with $N_{mass}$ * $P_{mass}$ interaction, with a colour grid for the overall trend. The response surface is $J_{max\_mass}$ = exp(5.535 + 0.388*ln($N_{mass}$) - 0.436*ln($P_{mass}$) + 0.295*(ln($N_{mass}$) * ln($P_{mass}$))), $r^2$ = 0.47 (Table 2). All variables in panels (a), (b) are based on natural logarithms, and the physiological capacities ($V_{cmax\_mass}$ and $J_{max\_mass}$) are normalised to 25 °C. Colour band scale for $V_{cmax\_mass}$ and $J_{max\_mass}$ rates are indicated.

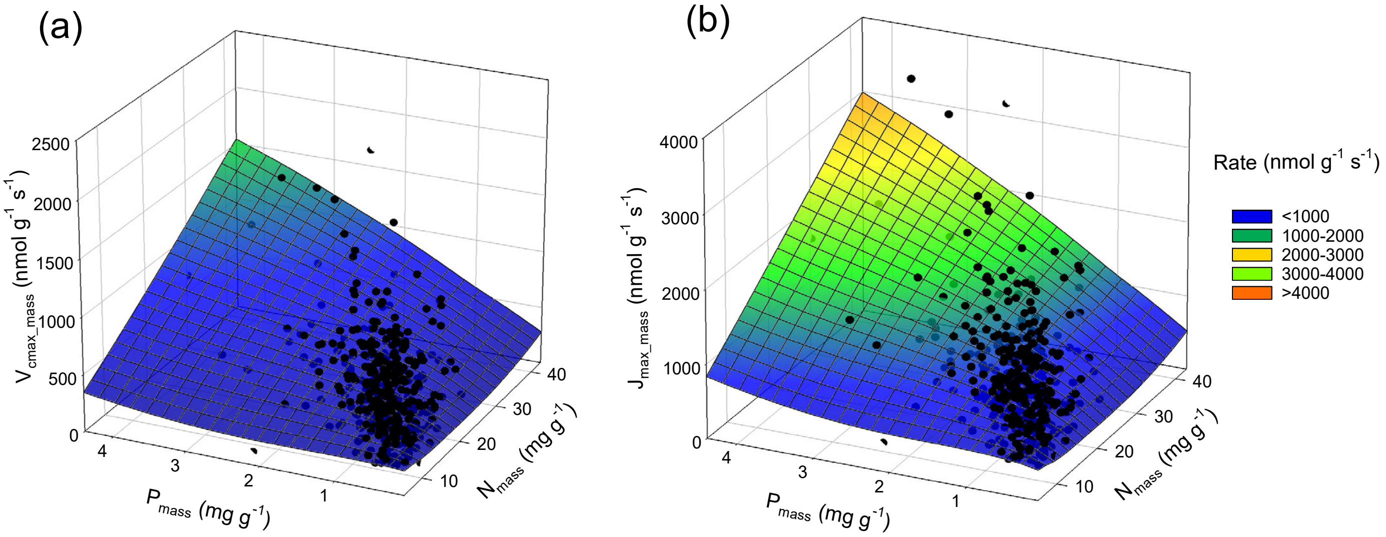

**Supplementary Figure 9. Modelled gross primary productivity (GPP) (averaged over 2001-2009) compared with different observation sets.**

Modelled GPP from the ORCHIDEE-CNP model is compared with observation-based and model-based for GPP with P constraints (GPP, from Fig. 4b) and without P constraints ($GPP_{pot}$; from Fig. 4a) employed. The various estimators used were: MODIS-estimated GPP is a main part of the MODIS product MOD17 (MODIS GPP, panels A, D), GPP fluxes derived from empirical up-scaling of eddy covariance measurement (MTE GPP. Panels B, E), and the Breathing Earth System Simulator model (BESS GPP, panels C, F). The colour scale shows the natural logarithm of point density, with darker shades representing more points. The black line indicates the 1:1 line.

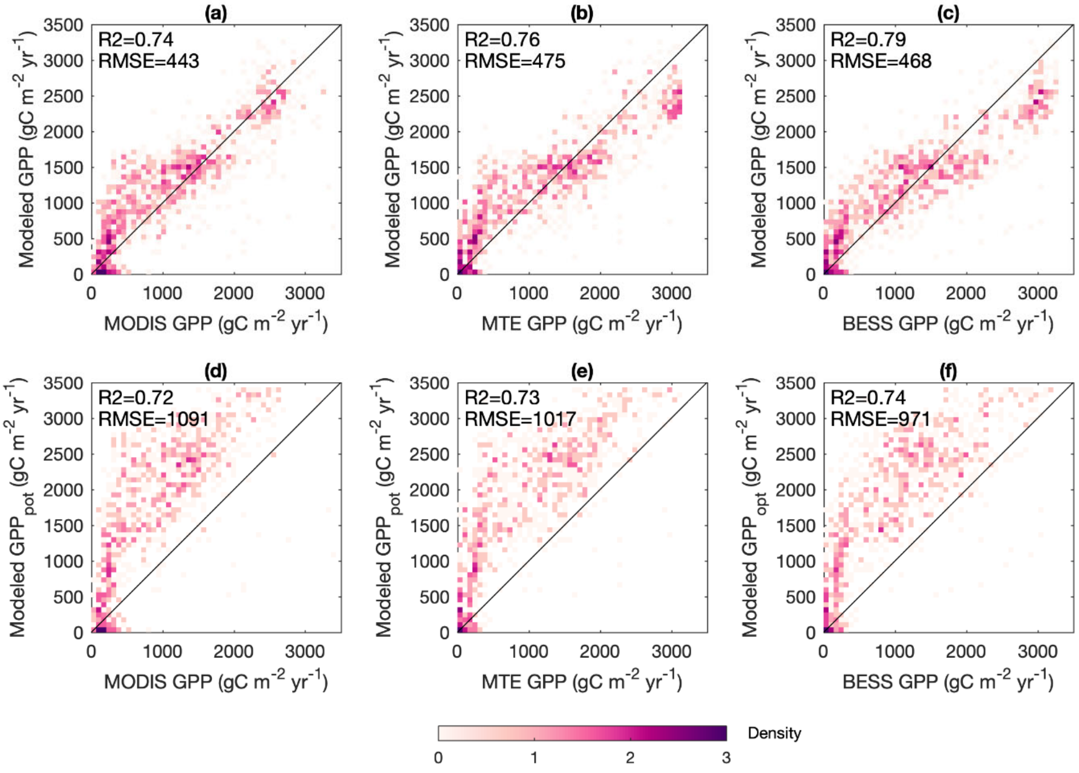

**Supplementary Figure 10. Framework for inclusion of data into the current study in the form of a PRISMA flowchart.** A report is defined here as a source (database, publication or person with data holding) with information on the group of species measured at a particular site.

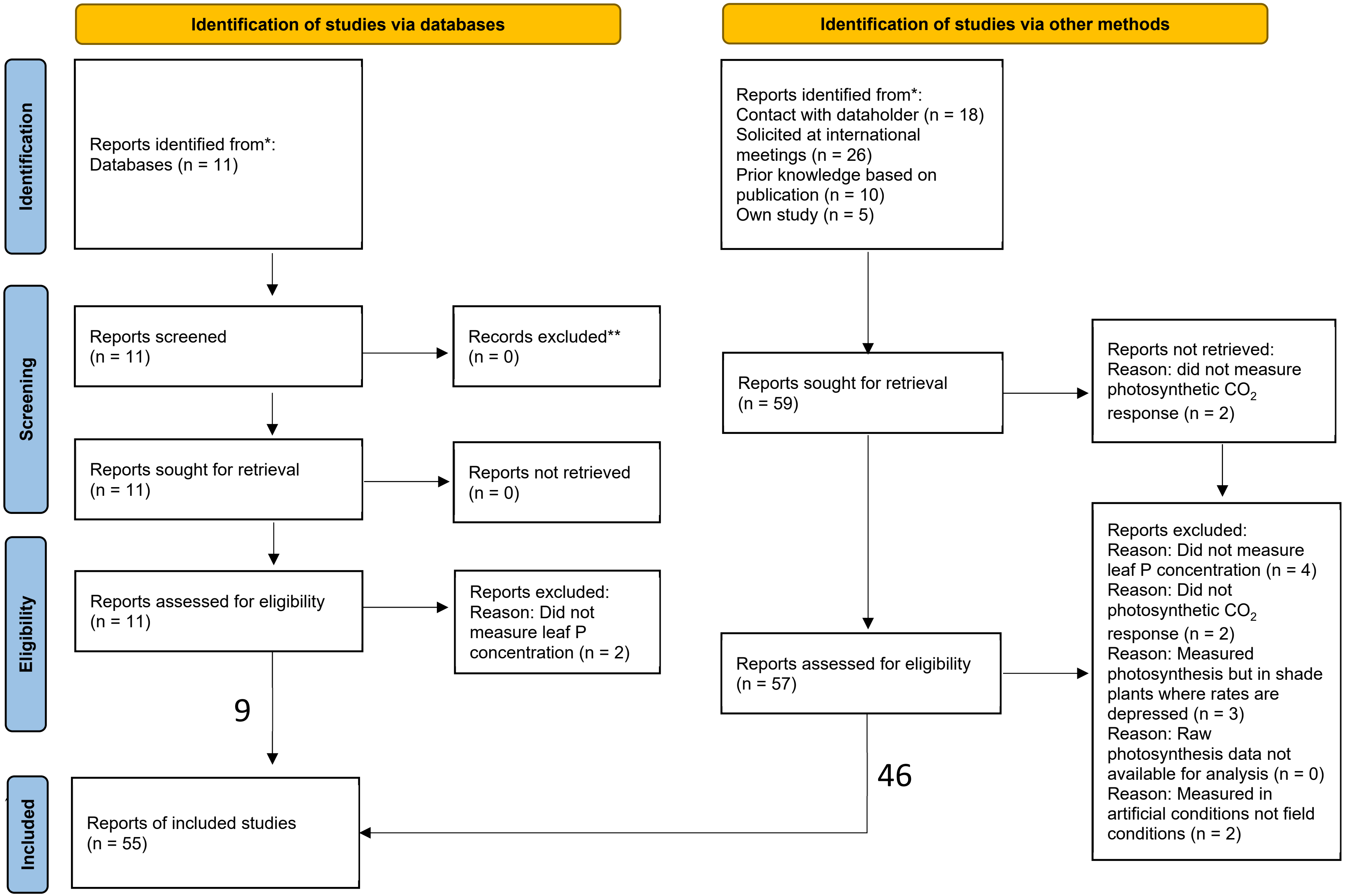

**Supplementary Figure 11. Sources of variance for leaf N and P concentrations ($N_{mass}$ and $P_{mass}$) in the data.**

Variance was partitioned according to the taxonomic classification, with residual indicated the unaccounted error.

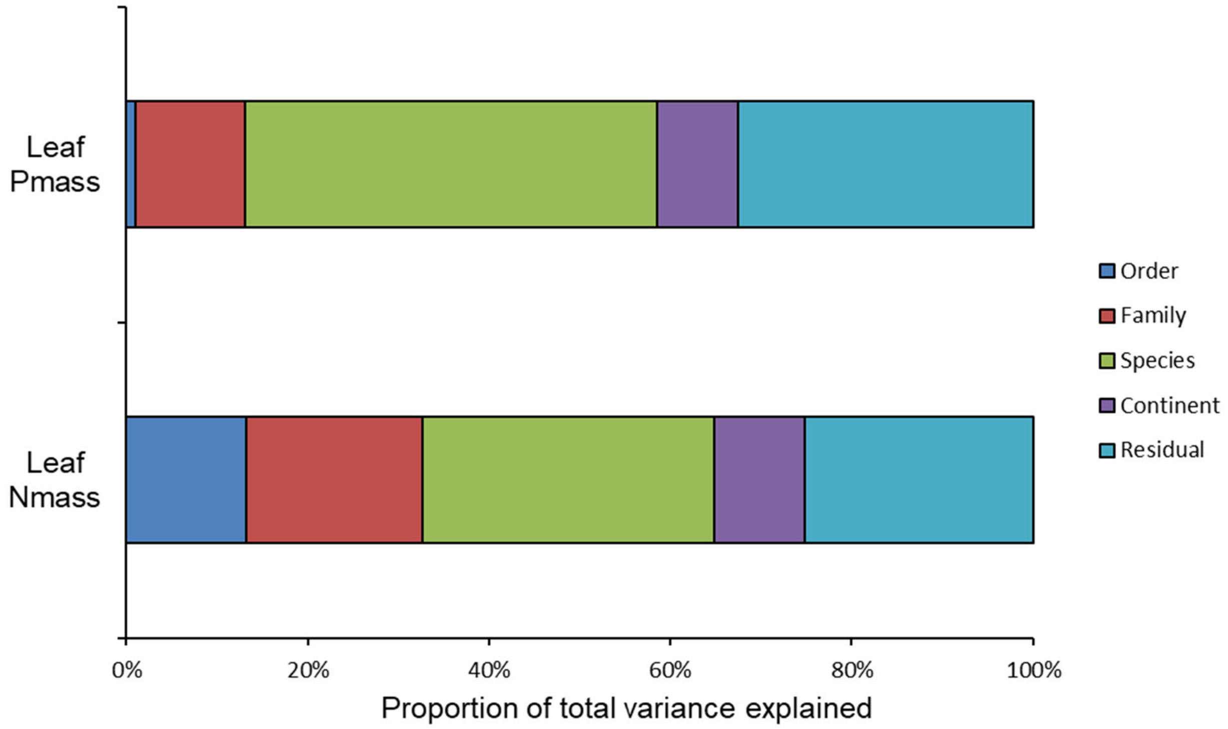

**Supplementary Table 1. Description of the study sites, climate parameters and data sources.**

The description includes climate parameters for the sites, number of species measured (number of species), mean measurement leaf temperature (leaf temperature) and data sources. Locations closer than 5 km to one another are aggregated into a single site. Elev. indicates the site elevation above sea level. For mean annual temperature and precipitation (precip.), when site-specific data were absent, values from global gridded meteorology (Worldclim v.2.1[73]) were used instead (indicated by †). When two sets of measurement temperatures are indicated, they denote two different investigators working at the same site in different campaigns in different years. Data sources are for the raw data for photosynthesis and leaf nutrients N and P. In some instances, both N and P were not measured for the same set of leaves and therefore those species were dropped from the final analyses.

| Site Name | Country | Elev. (m) | Mean annual temp (°C) | Mean annual precip. (mm) | # Species measured | Leaf temper-ature (°C) | Data source |
|---|---|---|---|---|---|---|---|
| Adelaide River | Australia | 75 | 27.0 | 1532 | 2 | 32.5 | [62] |
| Allpahuayo-site A | Peru | 126 | 26.3 | 2760 | 11 | 30.3 | [63] |
| Allpahuayo-site B | Peru | 150 | 25.2 | 2690 | 20 | 29.0 | [63] |
| Asukese | Ghana | 228 | 27.5† | 1271 | 4 | 30.2 | [29] |
| Bissiga | Burkina Faso | 275 | 28.3 | 897 | 7 | 34.6 | [29] |
| Boabeng-Fiema | Ghana | 360 | 25.4 | 1290 | 21 | 33.4 | [29] |
| Boulia | Australia | 151 | 24.9 | 291 | 2 | 33.7 | [62] |
| Bubeng | China | 682 | 23.4 | 1513 | 18 | 27.3 | Ellsworth, Zhang and Zhang |
| Cape Tribulation | Australia | 40 | 25.2 | 3200 | 19 | 28.5, 30.1 | [62], and Crous and Ellsworth |
| Caxiuana | Brazil | 1 | 25.7 | 2272 | 8 | 31.6 | [64] |
| Cuzco Amazonica | Peru | 205 | 24.4 | 1900 | 8 | 29.9 | [63] |
| Daly River | Australia | 73 | 27.2 | 1170 | 2 | 33.9 | [62] |
| Dano | Burkina Faso | 293 | 28.1† | 1017 | 6 | 35.0 | [29] |
| Davies Park | Australia | 390 | 17.1 | 1023 | 4 | 27.4 | [25], and Ellsworth |
| Davis Creek | Australia | 668 | 21.2 | 1450 | 3 | 30.2 | [62] |
| Dry Creek | Australia | 167 | 27.1 | 958 | 2 | 32.9 | [62] |
| Edmond Kennedy Park | Australia | 2 | 23.8† | 1970† | 3 | 34.9 | [62] |
| Esperanza | Peru | 2863 | 13.1 | 1560 | 5 | 27.0 | [63] |
| EucFACE/Hawkes-bury | Australia | 23 | 17.4 | 760 | 4 | 27.3 | [25] |
| FLONA-Km67 | Brazil | 89 | 25.0 | 1920 | 6 | 30.9 | [65] |
| FLONA-Km83 | Brazil | 100 | 26.8† | 1990† | 3 | 30.3 | [65] |
| Forty Mile Scrub | Australia | 748 | 21.7† | 830† | 5 | 32.8 | [62] |
| Hombori | Mali | 310 | 29.9 | 350 | 3 | 34.9 | [29] |
| Howard Springs | Australia | 37 | 27.8 | 1714 | 2 | 32.5 | [62] |
| Illawarra fly | Australia | 710 | 12.7 | 1849 | 2 | 24.8 | [25] |
| Jenaro Herrera | Peru | 124 | 26.6 | 2700 | 28 | 28.8 | [63] |

| | | | | | | | |
|---|---|---|---|---|---|---|---|
| Jurien Bay | Australia | 24 | 19.0 | 536 | 3 | 26.3 | Ellsworth, [25] |
| Kampong Thom | Cambodia | 117 | 27.4† | 1640† | 7 | 31.3 | [66] |
| Kauri Creek | Australia | 813 | 20.5 | 1960 | 8 | 27.4 | [62] |
| Kogyae Strict Nature Reserve | Ghana | 201 | 26.3 | 1250 | 8 | 34.5 | [29] |
| Koombooloomba | Australia | 860 | 19.8 | 1750 | 22 | 27.3 | [62] |
| Kosnipata | Peru | 3025 | 11.1 | 1706 | 8 | 21, 26.7 | [63,67] |
| Kratie | Cambodia | 96 | 26.9† | 1780† | 5 | 32.1 | [66] |
| Kuring-gai-Chase | Australia | 140 | 18.2 | 1400 | 2 | 27.1 | Ellsworth and Crous |
| Lambir Hills | Malaysia | 200 | 27.0 | 2740 | 2 | 30.3 | [68] |
| Lesueur National Park | Australia | 80 | 18.8 | 521 | 2 | 26.0 | [25] |
| Manaus/Cuieiras reserve | Brazil | 130 | 26.7 | 2252 | 6 | 30.1 | Garcia, Domingues et al. |
| Mban-Djeren | Cameroon | 770 | 24.0 | 1620 | 48 | 30.9 | [69] |
| Mole | Ghana | 135 | 27.9 | 1030 | 5 | 35.5 | [29] |
| Nouragues | Fr. Guiana | 110 | 26.3 | 3000 | 23 | 30.8 | [70] |
| Nyungwe National Park | Rwanda | 2200 | 14.6 | 1879 | 6 | 20.2 | Dusenge and Uddling |
| Paracou | Fr. Guiana | 19 | 26.3 | 3150 | 9 | 31.3 | [70] |
| Parque Natural Metropolitano | Panama | 150 | 26.5 | 1740 | 21 | 29.9 | [32,71] |
| Parque Nacional San Lorenzo | Panama | 130 | 25.3 | 3300 | 21 | 32.8, 28.1 | [32,71] |
| Rishton Scrub | Australia | 238 | 23.5† | 720† | 14 | 32.8 | [62] |
| San Pedro | Peru | 1651 | 18.2 | 5030 | 24 | 27.4 | [63] |
| Sturt Plains | Australia | 228 | 26.8 | 672 | 2 | 34.2 | [62] |
| Sucusari | Peru | 117 | 26.2 | 2750 | 12 | 29.4 | [63] |
| Tambopata | Peru | 215 | 24.4 | 1900 | 6 | 29.8 | [63] |
| Tumbarumba | Australia | 1260 | 9.8 | 1417 | 1 | 26.1 | [72] |
| Trocha Union-site A | Peru | 1885 | 18.0 | 2470 | 16 | 24.5 | [63] |
| Trocha Union-site B | Peru | 3044 | 11.8 | 1780 | 8 | 23.3 | [63] |

**Supplementary Table 2. List of plant families from the different continents.** The list of families in the dataset has the number of species within each family for each continent in parentheses. All the species are angiosperms, and there was also one gymnosperm family in the dataset (Podocarpaceae).

| Continent | Families and numbers of species |
|---|---|
| Africa | Anacardiaceae (6), Annonaceae (2), Apocynaceae (3), Araliaceae (1), Bignoniaceae (1), Bixaceae (1), Bombacaceae (1), Cannabaceae (1), Celastraceae (2), Chrysobalanaceae (3), Combretaceae (13), Ebenaceae (1), Euphorbiaceae (3), Fabaceae (17), Irvingiaceae (1), Lamiaceae (1), Malvaceae (6), Meliaceae (6), Moraceae (7), Myristicaceae (2), Myrtaceae (2), Ochnaceae (1), Phyllanthaceae (4), Rosaceae (1), Rubiaceae (3), Salicaceae (3), Sapindaceae (1), Sapotaceae (6), Simaroubaceae (1), Ximeniaceae (1) |
| Asia | Anacardiaceae (1), Annonaceae (2), Burseraceae (1), Clusiaceae (2), Combretaceae (3), Dipterocarpaceae (5), Elaeocarpaceae (1), Euphorbiaceae (1), Fabaceae (1), Fagaceae (3), Lardizabalaceae (1), Lauraceae (1), Malvaceae (1), Moraceae (3), Myristicaceae (1), Myrtaceae (3), Rubiaceae (3), Sapindaceae (1), Sonneratiaceae (1), Tiliaceae (1), Verbenaceae (1) |
| Australia | Altingiaceae (1), Anacardiaceae (1), Apocynaceae (2), Araliaceae (1), Boraginaceae (1), Casuarinaceae (1), Celastraceae (2), Combretaceae (1), Cunoniaceae (1), Elaeocarpaceae (3), Euphorbiaceae (3), Fabaceae (5), Lamiaceae (1), Lauraceae (8), Loganiaceae (1), Malvaceae (1), Monimiaceae (2), Myristicaceae (1), Myrtaceae (33), Oleaceae (1), Proteaceae (5), Rhamnaceae (2), Rutaceae (2), Sapindaceae (3), Sapotaceae (1) |
| South America | Anacardiaceae (4), Annonaceae (5), Apocynaceae (7), Aquifoliaceae (2), Araliaceae (3), Asteraceae (2), Bignoniaceae (1), Bixaceae (1), Boraginaceae (2), Brunelliaceae (1), Burseraceae (7), Calophyllaceae (1), Cardiopteridaceae (1), Caryocaraceae (2), Celastraceae (1), Chloranthaceae (1), Chrysobalanaceae (9), Clethraceae (4), Clusiaceae (4), Combretaceae (3), Convolvulaceae (2), Cunoniaceae (1), Dichapetalaceae (1), Elaeocarpaceae (3), Euphorbiaceae (8), Fabaceae (18), Goupiaceae (1), Hippocrateaceae (1), Humiriaceae (2), Lauraceae (15), Lecythidaceae (12), Lepidobotryaceae (2), Linaceae (2), Malpighiaceae (3), Malvaceae (9), Melastomataceae (5), Meliaceae (3), Menispermaceae (2), Moraceae (14), Myricaceae (1), Myristicaceae (5), Myrtaceae (1), Nyctaginaceae (1), Ochnaceae (2),Olacaceae (4), Opiliaceae (1), Pentaphylacaceae (1), Phyllanthaceae (2), Primulaceae (3), Proteaceae (1), Rhamnaceae (1), Rosaceae (1), Rubiaceae (6), Sapindaceae (1), Sapotaceae (24), Simaroubaceae (2), Styracaceae (1),Ulmaceae (1), Urticaceae (5), Vochysiaceae (2) |

**Supplementary Table 3. Standardised major axis (SMA) analysis**.

SMA is shown for photosynthesis and biochemical parameters and leaf N on an area and mass basis. Moderate (Mod.) and low P are defined in the text and in Fig. 1. $V_{cmax}$, $J_{max}$ and their mass-based equivalents are the rates normalised to 25 °C using kinetic constants, while $A_{net}$ and $A_{mass}$ are not adjusted for leaf temperature. Statistical probabilities (*P*-values) that are significant or significantly different by P status are given in bold. Where $P > 0.10$, not significant is denoted n.s.

| Response variable | Bivariate variable | P status | d.f. | $r^2$ | P-value | Intercept | Standardised slope | *P*-value for diff. in slope | *P*-value for diff. in intercept |
|---|---|---|---|---|---|---|---|---|---|
| Mass-based | | | | | | | | | |
| $A_{mass}$ | $N_{mass}$ | Mod. P | 233 | 0.23 | **<0.0001** | 1.628 | 1.626 | **0.015** | n.s. |
| | | Low P | 214 | 0.08 | **<0.0001** | 1.316 | 1.307 | | |
| $V_{cmax\_mass}$ | $N_{mass}$ | Mod. P | 233 | 0.26 | **<0.0001** | 1.445 | 1.456 | 0.074 | n.s. |
| | | Low P | 214 | 0.08 | **<0.0001** | 1.318 | 1.294 | | |
| $J_{max\_mass}$ | $N_{mass}$ | Mod. P | 233 | 0.23 | **<0.0001** | 1.390 | 1.399 | 0.063 | n.s. |
| | | Low P | 214 | 0.10 | **<0.0001** | 1.182 | 1.165 | | |
| Area-based | | | | | | | | | |
| $A_{net}$ | N | Mod. P | 237 | 0.001 | n.s. | - | - | n.s. | n.s. |
| | | Low P | 217 | 0.003 | n.s. | - | - | | |
| $V_{cmax}$ | N | Mod. P | 233 | 0.11 | **<0.0001** | 1.279 | 1.253 | n.s. | **0.021** |
| | | Low P | 214 | 0.07 | **0.00014** | 1.161 | 1.493 | | |
| $J_{max}$ | N | Mod. P | 233 | 0.10 | **<0.0001** | 1.599 | 1.206 | n.s. | **0.020** |
| | | Mod. P | 214 | 0.06 | **0.0005** | 1.538 | 1.270 | | |

**Supplementary Table 4.** Comparison of $V_{cmax\_mass}$-$P_{mass}$ and $J_{max\_mass}$-$P_{mass}$ slopes (Fig. 2) across different continents in this study. Mean slopes for each continent for these two relationships are shown, with 95% confidence intervals for these slopes in parentheses.

| Continent | $V_{cmax\_mass}$-$P_{mass}$ slope | $J_{max\_mass}$-$P_{mass}$ slope |
|---|---|---|
| Africa | 0.583 (0.372-0.793) | 0.580 (0.391-0.769) |
| Asia | 0.781 (0.541-1.022) | 0.655 (0.440-0.870) |
| Australia | 0.210 (0.074-0.345) | 0.336 (0.209-0.462) |
| S. America* | 0.597 (0.506-0.689) | 0.540 (0.457-0.623) |

*includes Central American tropics
