# Supplementary material for: Convergence in phosphorus constraints to photosynthesis in forests around the world
Source: Nat Commun. 2022 Aug 25;13:5005. doi: 10.1038/s41467-022-32545-0 (PMC9411118; doi:10.1038/s41467-022-32545-0)
Supplement: Supplementary file 3 — Reporting Summary [file 41467_2022_32545_MOESM3_ESM.pdf]

nature research

Corresponding author(s): Ellsworth

Last updated by author(s): Jul 27, 2022

Reporting Summary

Nature Research wishes to improve the reproducibility of the work that we publish. This form provides structure for consistency and transparency in reporting. For further information on Nature Research policies, see Authors & Referees and the Editorial Policy Checklist .

Statistics

For all statistical analyses, confirm that the following items are present in the figure legend, table legend, main text, or Methods section.

n/a	Confirmed	
☐	☒	The exact sample size (*n*) for each experimental group/condition, given as a discrete number and unit of measurement
☐	☒	A statement on whether measurements were taken from distinct samples or whether the same sample was measured repeatedly
☐	☒	The statistical test(s) used AND whether they are one- or two-sided<br>*Only common tests should be described solely by name; describe more complex techniques in the Methods section.*
☐	☒	A description of all covariates tested
☐	☒	A description of any assumptions or corrections, such as tests of normality and adjustment for multiple comparisons
☐	☒	A full description of the statistical parameters including central tendency (e.g. means) or other basic estimates (e.g. regression coefficient) AND variation (e.g. standard deviation) or associated estimates of uncertainty (e.g. confidence intervals)
☐	☒	For null hypothesis testing, the test statistic (e.g. *F*, *t*, *r*) with confidence intervals, effect sizes, degrees of freedom and *P* value noted<br>*Give P values as exact values whenever suitable.*
☒	☐	For Bayesian analysis, information on the choice of priors and Markov chain Monte Carlo settings
☐	☒	For hierarchical and complex designs, identification of the appropriate level for tests and full reporting of outcomes
☒	☐	Estimates of effect sizes (e.g. Cohen's *d*, Pearson's *r*), indicating how they were calculated

Our web collection on statistics for biologists contains articles on many of the points above.

Software and code

Policy information about availability of computer code

Data collection	Portable photosynthesis data were collected using Li-Cor Li-6400 software 'OPEN' v4.2, v.5, and v6.1.4.
Data analysis	R version 4.1.2 was used for all data analyses. The code for analyses of leaf data is available at https://github.com/ellswor2/photo_p_repo2.git. The plantecophys R package used was v1.4-6. The model code is at https://doi.org/10.14768/20200407002.1

For manuscripts utilizing custom algorithms or software that are central to the research but not yet described in published literature, software must be made available to editors/reviewers. We strongly encourage code deposition in a community repository (e.g. GitHub). See the Nature Research guidelines for submitting code & software for further information.

Data

Policy information about availability of data

All manuscripts must include a data availability statement. This statement should provide the following information, where applicable:

- Accession codes, unique identifiers, or web links for publicly available datasets
- A list of figures that have associated raw data
- A description of any restrictions on data availability

The complete dataset of leaf measurements generated during and/or analysed during the current study is available at https://doi.org/10.6084/m9.figshare.20010485.v1. The TRY database used was extracted from TRY v.5 at https://www.try-db.org/TryWeb/Home.php. The model results are archived at https://doi.org/10.5281/zenodo.661961.

Field-specific reporting

Please select the one below that is the best fit for your research. If you are not sure, read the appropriate sections before making your selection.

Ecological, evolutionary & environmental sciences study design

All studies must disclose on these points even when the disclosure is negative.

Study description	This is a data compilation of measurements of the photosynthetic CO_2 response under controlled conditions, alongside leaf chemical characteristics and morphology. We gathered published and unpublished data on controlled photosynthetic responses to [CO_2] for a set of pan-tropical and subtropical sites in Africa, Asia, Australia and South America. Site latitude was generally <35 ° N and S, with exception of the temperate Northern Hemisphere data in Supplementary Figure 7 that involved sites >33 ° N.
Research sample	The main dataset was comprised of 455 species, with a variable number sampled at any one of the 52 study sites in Supplementary Table 1 and geospatially depicted on the map in Supplementary Figure 2. The selection of data for inclusion was specified in Supplementary Figure 10. At each site, one leaf from each of 3-12 random individuals of each species were selected and measured, except for specific South American sites (Allpahuayo-site A, Allpahuayo-site B, Cuzco Amazonica, Esperanza, Jenaro Herrera, Kosnipata, San Pedro, Sucusari, Tambopata, Trocha Union-site A, and Trocha Union-site B) where only one individual per species was measured. Each species sampled was designated to represent the photosynthetic behaviour of a species at a particular site.
Sampling strategy	At each site, the researchers sampled common or abundant species within the constraints imposed by access to the upper part of the forest canopy, or sunlit leaves. Sample sizes at each site varied according to logistic considerations, as many of the field sites were remote and parts of the terrain were inaccessible. The overall sample size was designated to be as large as possible but with over 150 species representing both high and low P, as sample sizes larger than 150 species have not been previously assembled for this kind of data. Species samples > 150 species give more reliable results with greater precision and power.
Data collection	At each site, the researchers used the Licor model 6400 portable photosynthesis system to record data on the leaf photosynthetic response to CO_2 concentration changed step-wise, whilst controlling light and temperature. Light levels were constant and at levels saturating photosynthesis. Data were only logged manually or automatically when measurements were judged to be stable. Each user of the Licor model 6400 instrument had been trained and had read the user-manual for the instrument. In every case the data collectors were the manuscript authors or their direct, designated associates.
Timing and spatial scale	Data comprise samples collected between 1999 and 2019 depending on the investigator and site. The spatial extent of each site was approximately within 200 to 500 m^2 area.
Data exclusions	Within the selection constraints for the researchers working at each field site, we removed data where stomatal conductance was very low (stomatal conductance < 30 mmol H_2O m^{-2} s^{-1}) or mass-based Anet was exceptionally low (< 20 nmol CO_2 g^{-1} s^{-1}). Data were further excluded if the coefficient of variation for the fits for Vcmax was larger than 30%, indicating poor fits. Some of the field measurements involved photosynthetic CO_2 response curves that did not saturate (did not reach sufficiently high CO_2 concentrations inside the leaf) and hence the fitting algorithm could not converge. When this occurred these data (25 additional species) could not be used in the final analysis.
Reproducibility	Data are reproducible, and code for fitting the parameters that were analysed is publicly-available (plantecophys package v.1.4-6 in R), and also available.
Randomization	Randomisation was only relevant for the analysis in Figure 1. For this, we grouped data according to a previously-published threshold of P concentration (Reich et al. 2009 reference). We also tested alternative P groupings based on different thresholds and got similar results (Supplementary Figure 5). As this threshold increased in P concentration, the slope attributable to the low P grouping increased, commensurate with the inclusion of P as a another variable in the model.
Blinding	For the chemical analyses, standards and blind standards were submitted and were within a few percent of the known value. However, we accepted chemical analysis values from data contributors according to their own quality control and blinding approaches. This included samples run blind because they were identifiable only according to sequential sample numbers.

Did the study involve field work? ☒ Yes ☐ No

Field work, collection and transport

Field conditions	Information on site elevation, mean annual temperature and mean annual precipitation of all sites are provided in Supplementary Table 1 in Supplementary Material. Measurements of photosynthesis were made at light saturation and field temperatures ranging 20-35 degrees C with mean temperatures reported in Supplementary Table 1 (standard errors within sites were typically < 2.5 degrees C). The biochemical parameters Vcmax and Jmax were normalised to 25 degrees C for presentation according to Kumarathunge et al. (2019).
Location	Location names, countries and latitude, longitude of field sites are specified in Supplementary Table 1 in Supplementary Material
Access and import/export	Each researcher who contributed data was responsible for their own efforts to access habitats without damage, and to collect and import/export plant samples in a responsible manner. When required such as for sampling within national parks, the appropriate permits were obtained by the responsible investigator for those samples. In such cases, permit numbers are available upon request to the researcher who provided the data from each location.
Disturbance	For some sites, canopy access was not possible so cut branches were taken. The branch cutting was distributed in a fashion that would not damage the specific forest area being sampled.

Reporting for specific materials, systems and methods

We require information from authors about some types of materials, experimental systems and methods used in many studies. Here, indicate whether each material, system or method listed is relevant to your study. If you are not sure if a list item applies to your research, read the appropriate section before selecting a response.

Materials & experimental systems

n/a	Involved in the study
☒	☐ Antibodies
☒	☐ Eukaryotic cell lines
☒	☐ Palaeontology
☒	☐ Animals and other organisms
☒	☐ Human research participants
☒	☐ Clinical data

Methods

n/a	Involved in the study
☒	☐ ChIP-seq
☒	☐ Flow cytometry
☒	☐ MRI-based neuroimaging
